# Elastic Coupling of Nascent apCAM Adhesions to Flowing Actin Networks

**DOI:** 10.1371/journal.pone.0073389

**Published:** 2013-09-06

**Authors:** Cecile O. Mejean, Andrew W. Schaefer, Kenneth B. Buck, Holger Kress, Alla Shundrovsky, Jason W. Merrill, Eric R. Dufresne, Paul Forscher

**Affiliations:** 1 Department of Mechanical Engineering and Materials Science, Yale University, New Haven, Connecticut, United States of America; 2 Department of Molecular, Cellular and Developmental Biology, Yale University, New Haven, Connecticut, United States of America; 3 Department of Physics, Yale University, New Haven, Connecticut, United States of America; 4 Department of Chemical and Environmental Engineering, Yale University, New Haven, Connecticut, United States of America; 5 Department of Cell Biology, Yale University, New Haven, Connecticut, United States of America; Dalhousie University, Canada

## Abstract

Adhesions are multi-molecular complexes that transmit forces generated by a cell’s acto-myosin networks to external substrates. While the physical properties of some of the individual components of adhesions have been carefully characterized, the mechanics of the coupling between the cytoskeleton and the adhesion site as a whole are just beginning to be revealed. We characterized the mechanics of nascent adhesions mediated by the immunoglobulin-family cell adhesion molecule apCAM, which is known to interact with actin filaments. Using simultaneous visualization of actin flow and quantification of forces transmitted to apCAM-coated beads restrained with an optical trap, we found that adhesions are dynamic structures capable of transmitting a wide range of forces. For forces in the picoNewton scale, the nascent adhesions’ mechanical properties are dominated by an elastic structure which can be reversibly deformed by up to 1 µm. Large reversible deformations rule out an interface between substrate and cytoskeleton that is dominated by a number of stiff molecular springs in parallel, and favor a compliant cross-linked network. Such a compliant structure may increase the lifetime of a nascent adhesion, facilitating signaling and reinforcement.

## Introduction

Adhesions are molecular assemblies connecting intracellular acto-myosin networks to external substrates. The mechanical coupling of substrate to cytoskeleton allows cells to sustain shape and facilitates directed movement via traction force production and protrusion. Integrin-based focal adhesions have been described as stratified plaques [1] composed of hundreds of proteins and regulatory molecules [2]. These complex structures are very dynamic. They typically assemble close to the leading edge of the cell, move in the direction of retrograde actin flow and eventually disassemble [3]. The level of engagement of adhesions to actin flow is believed to vary as adhesions age [3]. Previous studies have proposed three main phases for the development of adhesions that transmit forces to the substrate [4,5]. In the first stage, adhesions start to assemble but appear to remain uncoupled from the actin flow. Little or no force is transmitted to the substrate. Next, the newly formed adhesions intermittently couple to the actin flow [6]. An adhesion in this phase is sometimes referred to as a “slipping clutch”. Here, the forces are on the picoNewton scale and the actin flow is expected to be only weakly perturbed. The first two phases comprise what we have referred to previously as the “latency phase” in neuronal growth cones. In the final phase, nanoNewton scale forces can be transmitted to the external substrate and the local actin flow is strongly perturbed. Together, these processes allow adhesions to act as a “molecular clutch” [7], modulating the force transmitted to substrates.

The underlying physical mechanism of these three stages have been the subject of many models that incorporate stochastic binding and unbinding of stiff and short length scale spring-like connections between the substrate and the underlying actin network [8,9,10,11,12,13]. While these models have described trends of adhesion maturation with some success, they make specific assumptions about the mechanics of the interface between the substrate and the cytoskeleton that have not been tested. In addition, models have largely been developed with integrin-mediated adhesions in mind and less is known about the mechanical events underlying IgG superfamily adhesion formation.

ApCAM is an IgG superfamily cell adhesion molecule related to vertebrate NCAM that mediates homophilic cell-cell interactions such as neurite fasciculation [14,15]. In initial *in vitro* studies we found that *Aplysia* bag cell neuron growth cones exerted traction forces on neurites being used as fasciculation and growth substrates. During these interactions apCAM and actin filaments accumulated at sites of target contact and traction forces depended on actin filament assembly and retrograde actin flow [16]. These native growth cone-target interactions could be recapitulated using micro-beads coated with recombinant apCAM or an anti-apCAM antibody if beads were physically restrained to enable traction force generation [15]. Src family tyrosine kinase signaling and microtubule assembly dynamics were implicated in the mechano-transduction events leading to apCAM adhesion maturation [17,18]. These studies of acute interactions revealed an inverse correlation between rates of retrograde actin flow and rates of advance [15,16,18,19] in the growth cone, thereby strengthening the emerging “molecular clutch hypothesis” [18,20]. However, characterization of biomechanical events during the first or so-called “latency phase” of nascent adhesion formation remained to be addressed.

During the latency phase, retrograde flow rates remain unchanged. In some cases, local actin assembly occurs at the nascent adhesion site. This can generate cup-like actin structures surrounding apCAM beads or actin “comet tails” that stream off the distal side of restrained bead targets [15]. Biomechanical events during the latency phase have not been characterized. To address this outstanding problem, we designed experiments to correlate actin dynamics with forces measured during the latency phase of adhesion development. We report the presence of a mechanical connection between retrograde flow and apCAM beads that is softer and much larger than expected. Specifically, the effective spring constant is 10 to 100 times smaller and the typical length at failure is 100 to 1000 times greater than assumed in prevailing models.

## Materials and Methods

### Cell culture, substrate and beads preparation

*Aplysia* bag cell neurons were cultured on poly-L-lysine-coated coverslips in L15 medium supplemented with artificial seawater (ASW) (Life Technologies) [21]. The imaging medium was supplemented with 5 mg/ml BSA and free radical scavengers as previously described [19]. We coated 2 µm diameter polystyrene Ni-NTA beads (Micromod, Rostock-Warnemuende, Germany) with purified recombinant his-apCAM as previously reported for 5µm Ni-NTA silica beads [22]. Briefly beads were incubated at 1% (w/v) with purified his-apCAM (300 µg/ml) in PBS over-night at 4C. Residual sites were blocked with 5 mg/ml BSA PBS for 30 min and beads were stored in ligand solution. Beads were washed twice with PBS before diluting into imaging media for experiments. We compared the probability of binding the cell membrane for the bare Ni-NTA- and apCAM-coated beads to assess non-specific binding of the relatively hydrophobic polystyrene beads. All experiments were conducted in the presence of 5mg/ml BSA. The Ni-NTA-coated beads coupled 18% of the time and the apCAM-coated beads coupled 100% of the time.

### Attachment of 100nm fluorescent beads to 2µm apCAM-NiNTA beads

Suspensions of 100 nm diameter red fluorescent beads (0.2%) and apCAM-NiNTA beads (0.1%) were incubated together in 20 µl of ASW for about 20 minutes. The high concentration of ions in the buffer decreased the screening length between the beads and allowed them to adhere to one another when colliding under Brownian motion. The suspension was monitored under the microscope to asses when one fluorescent bead was attached on average to one apCAM bead. The suspension was then diluted by adding 500 µls of ASW and added to the tissue culture chamber.

### Live cell fluorescence

To visualize the actin filaments, a low concentration of Alexa 594-phalloidin appropriate for fluorescent speckle microscopy was injected into the cell as described [19].

### Micro-manipulation and optical microscopy

We used two different microscopy setups, one to measure the force-displacement curves of nascent adhesions and the other to correlate actin dynamics with trapped bead motion and to measure rotational motion. The first system combines holographic optical tweezers (HOT) with DIC and brightfield microscopy as previously described in [23]. The second system combines HOT with spinning disk confocal microscopy. Here, we used a spatial light modulator from Boulder Nonlinear Systems (P512-1064-HS, Lafayette, CO) and a 10 W 1064 nm wavelength CW laser from IPG Photonics (YLR-10-1064-LP, Oxford, MA). We used a Andor Revolution spinning disk confocal system (South Windsor, CT) mounted on a Nikon Ti inverted microscope (Melville, NY). For the visualization of actin and trapped beads, we used multi-mode imaging DIC/fluorescence. The beads were tracked in DIC using a cross-correlation algorithm and the actin was visualized in fluorescence using Alexa 594-phalloidin. For the measurement of the rotational motion of trapped beads, we simultaneously illuminated the sample with green fluorescent light to visualize the 100 nm red fluorescent beads (Invitrogen, Grand Island, NY) and with IR light to visualize the 2 µm apCAM-coated bead. Light coming from the sample was then separated by wavelength: wavelengths below 700 nm corresponding to fluorescent emission were sent to a Andor Ixon camera and wavelengths above 700 nm were sent to a Marlin F131B ASG NIR camera (Allied Vision Technology, Newburyport, MA).

### Trap stiffness calibration

Prior to each experiment, trapped apCAM-coated beads were held about 4 µm above the membrane of the growth cone for trap calibration. Images of the beads were acquired with an exposure time of 100 µs at 500 Hz over 2 min using a Photron 1024PCI fast camera (San Diego, CA). The centers of the beads were tracked over time using algorithms described in [24]. The distribution of positions was then fitted with a two-dimensional Gaussian function, which returned the covariance matrix. The covariance matrix was diagonalized to extract the principal axes, ${\hat{e}}_{1}$ and ${\hat{e}}_{2}$. The stiffnesses along the principal axes, *k*_trap,1_ and *k*_trap,2_, were then calculated using the equipartition theorem. This calibration was used to calculate the total force on the bead during experiments:$\vec{F}=\sum_{i}\left(k_{\text{trap},i}\Delta \vec{x}\cdot {\hat{e}}_{i}\right)\;\;{\hat{e}}_{i}$.

## Results and Discussion

**Figure 1:**
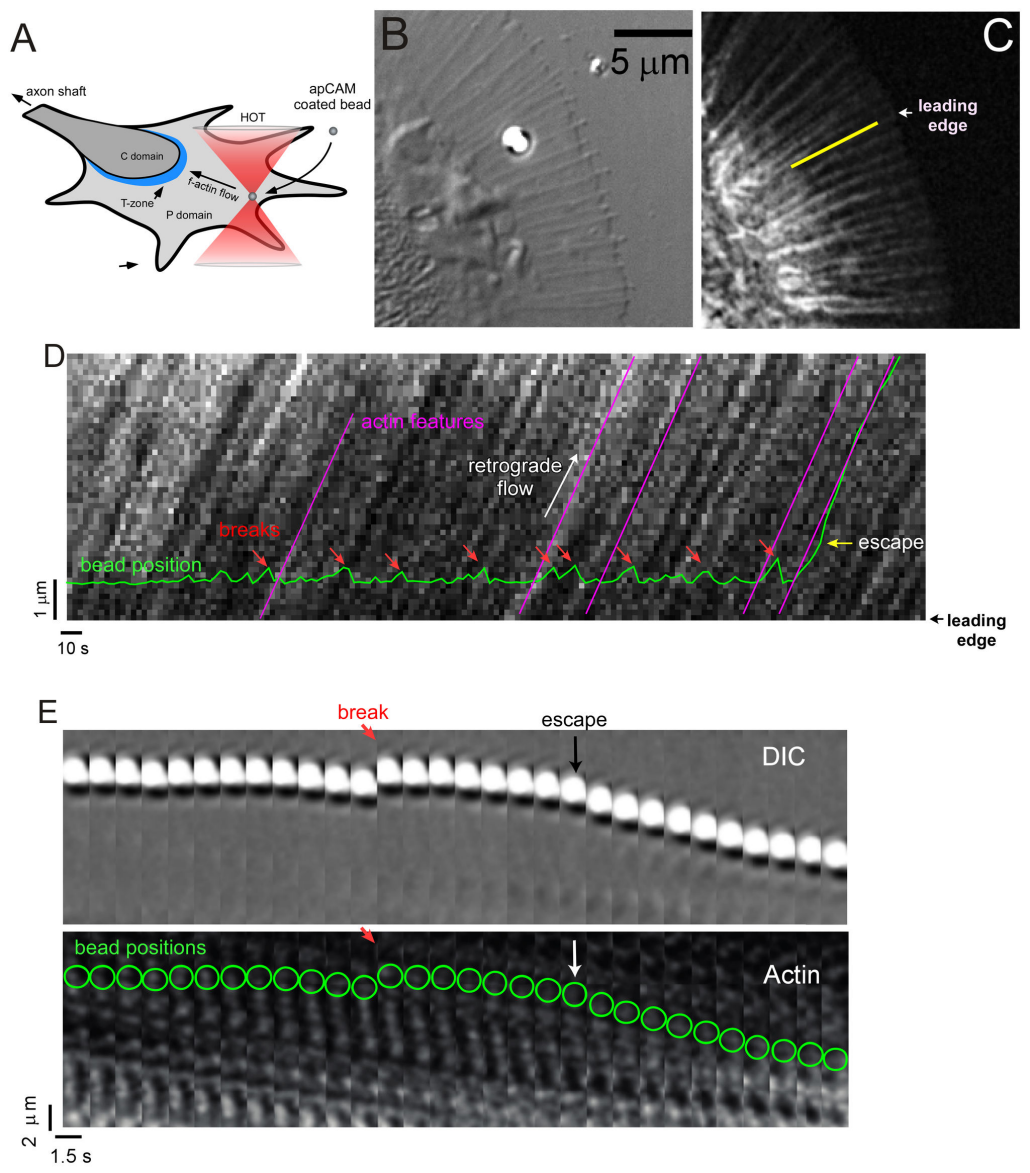
Correlation of bead movements with actin flow. (A) Schematic depicting growth cone cytoplasmic domains and an apCAM coated bead restrained against retrograde flow on the surface of an *Aplysia* bag cell growth cone using an optical trap. (B) DIC image of trapped apCAM-coated bead. (C) F-actin labeled with fluorescent phalloidin imaged with confocal microscopy. (D) Kymograph of actin speckle displacement sampled along the yellow line in (C). Green line shows trapped bead trajectory. Magenta lines show F-actin retrograde flow speed. Red arrows denote breakage events. The yellow arrow indicates the point of maximum optical force where the bead escapes from the trap. (E) Time-lapse montage DIC (top) and phalloidin-tagged actin (bottom) of a bead forming an adhesion, coupling to retrograde flow, a breakage event followed by recoupling to actin flow and eventual bead escape. Bead positions are superimposed on the phalloidin montage (green circles). Red arrow: breakage event; white/black arrow, bead escape. Time and space for C-D indicated by respective scale bars.

When apCAM coated beads are placed on the growth cone surface with an optical trap they rapidly couple to retrograde actin network flow (Figure 1A). To investigate the mechanism of apCAM coupling to the underlying actin network flow, we simultaneously recorded bead and F-actin dynamics (Figure 1B and C, Movie S1). The actin kymograph shown in (Figure 1D) shows that F-actin velocity (0.12 µm/s) was essentially uniform throughout the growth cone peripheral domain (Figure 1D magenta lines). The trapped beads exhibited small fluctuations and intermittent periods of steady movement (green line) in the direction of retrograde actin flow. Centripetal movements were followed by apparent linkage rupture events resulting in bead relaxation to the optical trap center (Figure 1D-E; red arrows). The observed intermittent formation and breakage of nascent adhesions is referred to as “stick-slip” behavior and has been reported for a variety of ligands and cell types using stationary optical tweezers [25,26]. While large centripetal bead displacements can be unambiguously identified as cytoskeletal coupling events, smaller displacements can be difficult to distinguish from thermal noise without imposing an arbitrary cut-off. Bead movement within the optical trap was slower than retrograde actin flow (Figure 1D, compare slopes of bead trajectory (green points) and actin flow (magenta lines)). When the bead finally escaped the trap, it initially jumped towards the central domain, faster than retrograde flow, and then moved steadily with retrograde flow (yellow arrow indicates the point of maximum force, beyond that point the force drops and vanishes about 2 µm away from the trap center). Intriguingly, the bead caught up with the same actin feature it was registered with at the time of initial bead movement. Another example is shown as a time-lapse montage in Figure 1E and in Figure S2. These observations suggested transient coupling to the cytoskeleton through elastic linkages.

**Figure 2:**
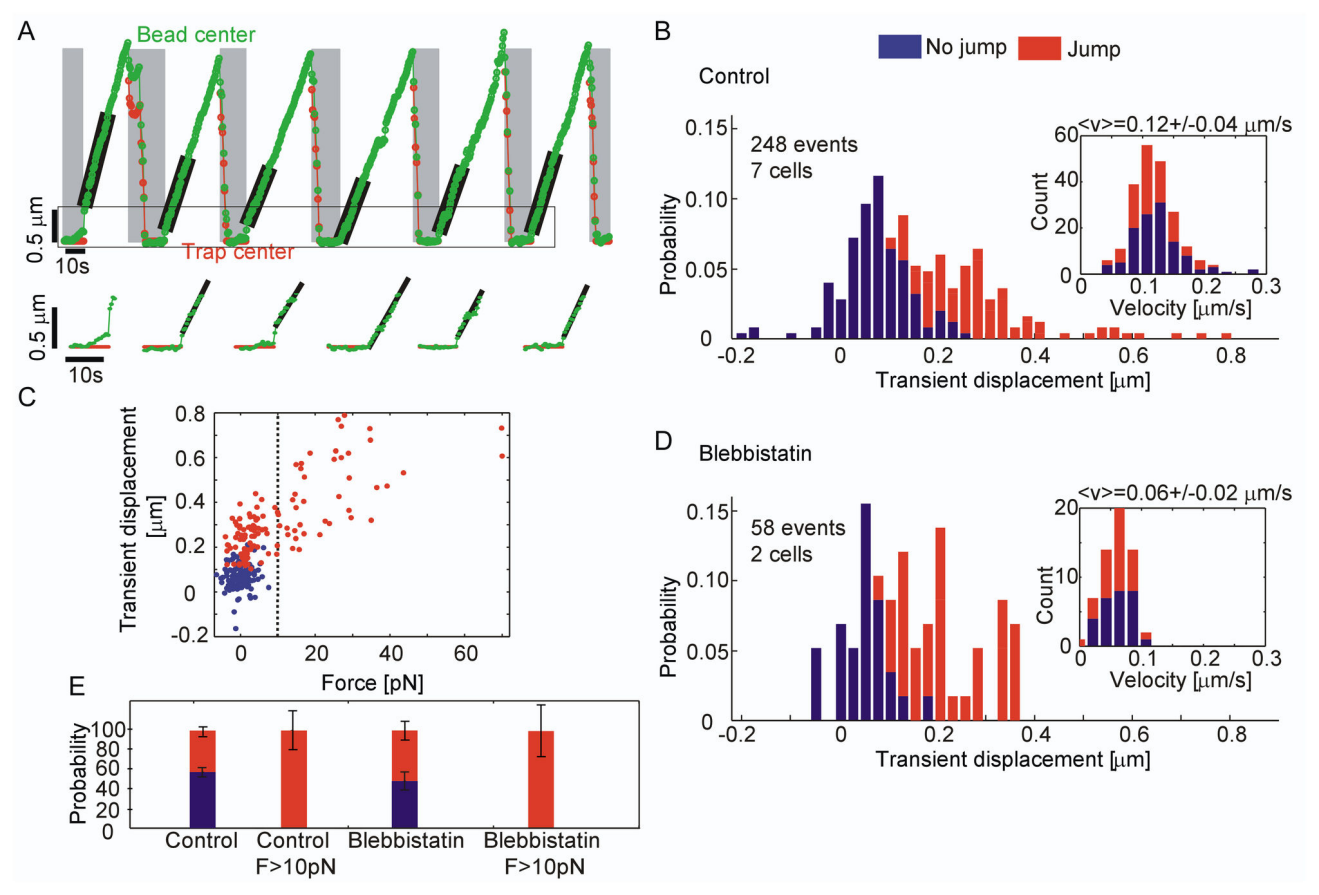
Relaxation of bead-substrates released from optical tweezers. (*A*) **apCAM-coated bead (green) is restrained 10 s in a stationary trap (red)**. The trap is then turned off and the bead moves with actin retrograde flow. A closed-loop trapping system returns the bead to its original position after it has been displaced by about 3 µm. Shaded and white areas represent periods where the optical tweezers are on and off, respectively. An enlargement showing bead displacement kinetics before and after release (boxed region) is shown below. (*B*) Histogram of transient displacements defined as the distance traveled by the bead during the first 0.8 s after turning laser off. Inset: histogram of the steady state bead velocity after each transient event: 0.8 s after release, the steady-state velocity was measured over the next 8 to 12 s. (*C*) Transient displacements versus force applied to beads just before turning off the laser. Note that 100% of beads exhibited elastic relaxation when force was >10 pN. (*D*) Histogram of transient displacement for a growth cone treated with 50 µM blebbistatin. Inset: histogram of the steady state bead velocity after each transient. (*E*) Bar graph showing the percentage of beads exhibiting no jump (blue), and the percentage exhibiting a jump (red), in control or blebbistatin-treated experiments. The first and third plots include all events after laser turn-off, while the second and fourth plots include events where the pre-stress exceeded 10 pN.

To investigate the apparent storage of elastic energy observed in Figure 1, we replaced a standard passive trap with an active trap controlled by a cyclic trap-and-release protocol. This assay allowed us to quantify the frequency of the different types of coupling and to resolve weaker events. We restrained beads with the optical tweezers for 5-20 s and then released them for ~30 s while their unrestrained motion was recorded. We then pulled the beads back to their initial position with a force clamp and repeated the cycle (Figure 2A, Movie S2). Figure 2 shows data for beads restrained for 10 sec, results for restraining times of 5 and 20 s show essentially the same results (Figure S1). When released from the optical trap, the beads almost always moved toward the central domain, in the direction of actin retrograde flow (Figure 2A). We fitted the steady motion after bead release to extract speeds, which are displayed as a histogram in the inset of Figure 2B. This is a unimodal distribution with a mean of 0.12 µm/s corresponding to typical retrograde flow speeds for *Aplysia* growth cones [27]. To characterize the transient motion of the bead upon release, we measured the bead displacements within the first 0.8 s after the laser was turned off. A histogram of the transient sizes is shown in Figure 2B. The peak around 100 nm corresponds to beads moving at the steady retrograde flow speed immediately upon release. However, we saw many events where the beads travelled much farther. Throughout Figure 2, we color-code events according to transient behavior. Blue bars represent events where the transient bead velocity did not significantly exceed the retrograde flow rate (we used a cutoff of the steady-state velocity plus the measurement error). Red bars indicate ‘jumps’, events where the transient velocity exceeded the retrograde actin flow rate. The largest displacements were all identified as jumps (Figure 2B) and the probability of a jump did not appear to depend on the retrograde flow speed (inset of Figure 2B). Figure 2C is a scatter plot of jump size versus pre-stress. Interestingly, the events with the largest transient displacements all had a significant pre-stress. There was a systematic increase in jump size with applied forces between 0 and 40 pN. The two data points near 70 pN suggest that the extension may saturate at larger forces.

Since retrograde flow speeds are known to depend on acto-myosin contractility, we treated the cells with blebbistatin, a specific inhibitor of the molecular motor non-muscle myosin II [28], and repeated the trap-and-release assay. We found the same basic behavior: upon release, all beads moved steadily towards the central domain, and some showed a small jump (Figure 2D). However, we found that the steady bead velocity dropped by a factor of two (inset Figure 2D), consistent with previous studies on *Aplysia* growth cones [29] where retrograde flow rates were attenuated by 30-50% under these experimental conditions. The transient sizes were significantly smaller (Figure 2D, compare red bars to Figure 2B). The main peak around 50 nm corresponds to bead motion at the mean retrograde actin flow rate during the first 0.8 s after release. Again, we found that events with a significant pre-stress show the largest jumps (Figure S1D).

We summarize these findings in Figure 2E. Even though assays with stationary optical traps show only intermittent bead motion (Figures 1-2), we found that upon release from the optical trap, beads were always coupled to retrograde actin flow, moving toward the central domain. In some cases, the bead rapidly jumped in the retrograde direction before settling down to the retrograde flow speed. Under control conditions, this happened 43% of the time. We observed no correlations between two successive events. Specifically, the probability of observing a jump in one event given that a jump was observed in the previous event was 46%. On the other hand, there was a perfect correlation between pre-stress and the observation of a jump: 100% of the events with a pre-stress above 10 pN showed a jump (Figure 2E Figure S1B). Reduced acto-myosin contractility with blebbistatin (Figure 2E) and varied restraining times had no significant impact on these probabilities (Figure 2E). Similarly, this probability did not change significantly when the time in the trap was varied from 5 to 20 seconds (Figure S1).

**Figure 3:**
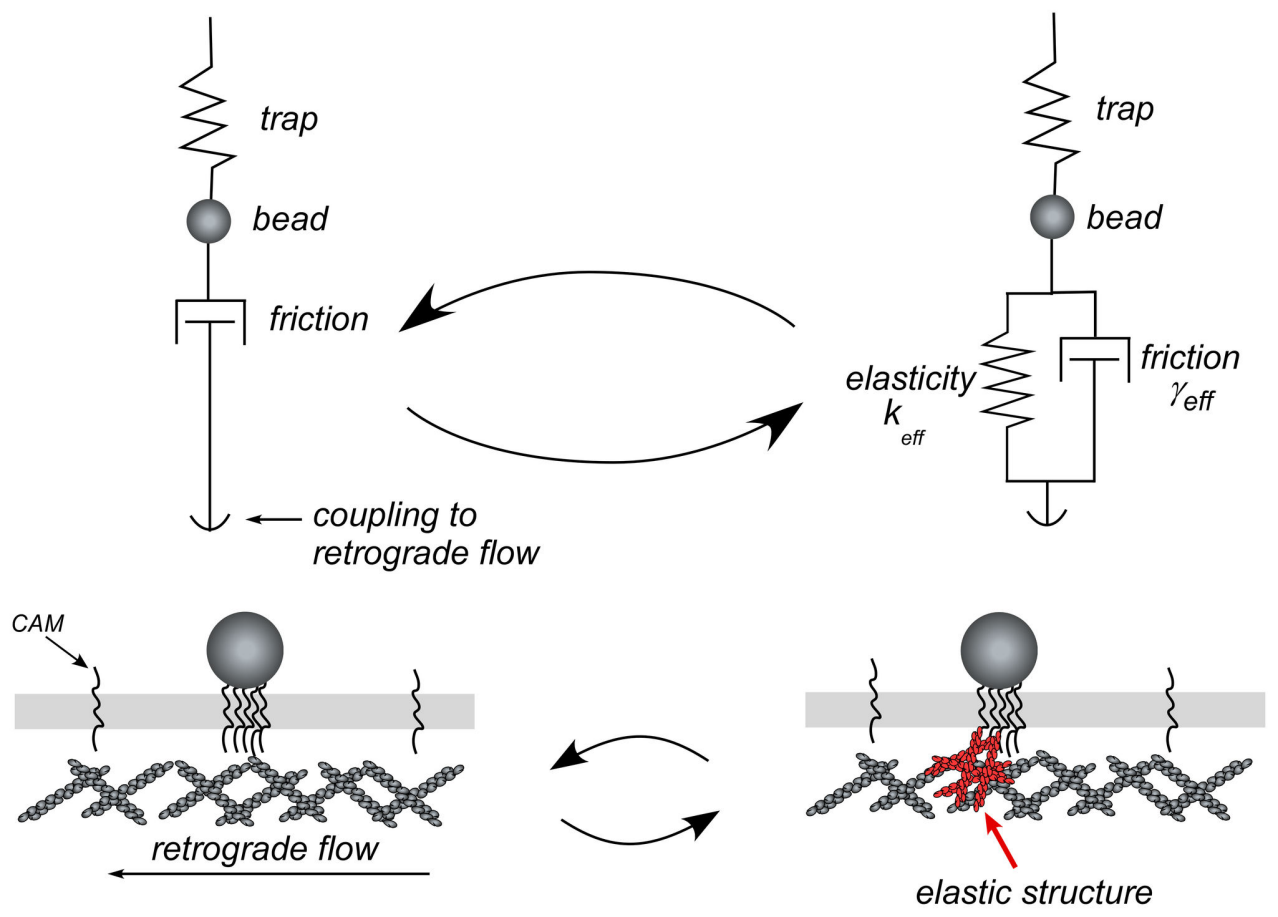
Schematic mechanical model of a nascent adhesion. The trapped bead alternates between frictional coupling with retrograde flow (left) and intermittent coupling to elastic intracellular structure (rignt).

The above data suggest that beads are coupled to the flowing actin via an intermittent elastic connection in parallel with a persistent viscous or ‘frictional’ component (Figure 3). The viscous drag between the bead and the cytoskeleton, characterized with the drag coefficient *γ*_*eff*_, is sufficient to translate a free bead at the retrograde flow rate but is too weak to displace a bead in an optical trap. Intermittent elastic connections, characterized by a spring constant *k*_*eff*_, are much stronger and can be sufficient to remove a bead from an optical trap. Along the way the elastic connection is stretched by the opposing forces of the optical trap and retrograde flow. When the bead escapes the trap limits (or the trap is turned off), the bead jumps forward, releasing the energy stored in the elastic connection (Figure 1D-E). The size of this jump depends on the amount that the connection was stretched. The connection is stretched more when the actin moves farther (Figure 2B and D) and the force applied by the optical trap is greater (Figure 2C). The following measurements were designed to test this emerging model and quantify the elastic and viscous properties of the nascent adhesions.

**Figure 4:**
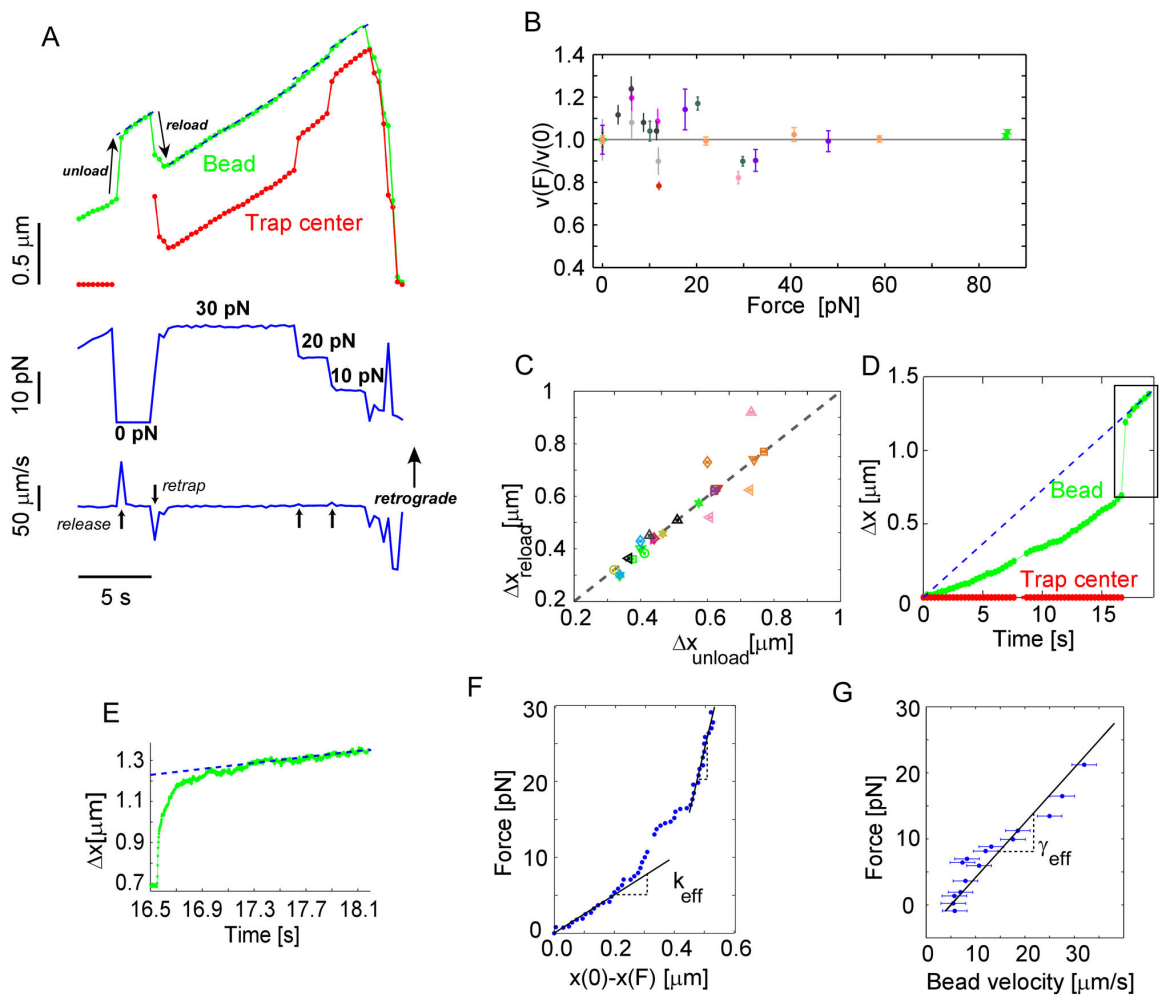
Elastic properties of nascent adhesions. (*A*) Top panel: Bead (green) vs. trap (red) relative positions. Flow-coupled apCAM-coated bead (green) is successively clamped at different constant forces by changing the separation between the bead and the center of the optical trap (red). Middle panel: flow-coupled bead is first released from the optical trap (“unload” arrow) and then force-clamped (“reload” arrow) at 30, 20 and 10 pN successively. Bottom panel: bead velocity exhibits a transient for each change of force indicated by the black arrows (B) Steady bead velocity under constant force normalized to bead velocity under zero force. Same color symbols correspond to same bead clamped at different forces. For each particular experiment the laser power of the trap is first set and then the distance between trap and bead was adjusted. (C) Jump sizes of flow-coupled beads when released from (Δ*x*_*unload*_) and reloaded by(Δ*x*_*reload*_) optical traps. Same color symbols correspond to different events with the same bead and cell. (D-E) When a flow-coupled bead (green) was displaced by 0.7 µm from the trap center (red), the bead was released. Dashed lines in D and E depict the extrapolated position of actin features moving with retrograde flow. (F) In-plane force-displacement curve of nascent adhesion. Displacement is defined as the distance between the bead and the blue dashed line in D representing the trajectory of an unrestrained flow-coupled bead. (G) Force-velocity of the flow-coupled bead during relaxation calculated using bead trajectory in *F* and force-displacement curve in *H*.

To verify that the elastic connection was bound to the flowing actin, we analyzed the motion of flow-coupled beads under a constant restraining force. This was a closed-loop measurement, where we maintained a constant vector separation between the centroid of the bead and the trap center by moving the trap based on bright-field images of the bead which were processed in real-time. We set the constant force value over a wide range by changing the laser intensity and the distance between the bead and the trap (top of Figure 4A and Movie S3). Under a constant force, the beads moved at a constant velocity (middle and bottom of Figure 4A). With each change in the restraining force, there was a transient spike in bead velocity as indicated by the black arrows. The value of the steady velocity did not change with the applied force and was equal to the retrograde flow velocity over the full range of forces accessible with the optical trap (Figure 4B). Here, the bead velocities were normalized to their values at zero force. Raw data for this experiment is shown in Figure S3. These results suggest that elastically coupled beads do not slip with respect to the retrograde flow. To more rigorously test for slip, we investigated the reversibility of the connection. We compared the jump size of a flow-coupled bead when it is unloaded by (released from) the optical trap, Δ*x*_*unload*_, to the displacement of the bead when it is reloaded at the same force, Δ*x*_*reload*_ (Figure 4C). This measurement was performed over a wide range of loading forces from 4 to 70 pN. The magnitude of Δ*x*_*unload*_ and Δ*x*_*reload*_ were essentially the same, showing that the deformation is reversible. This observation confirms the absence of slip between the elastic connection and the actin.

We quantified the effective spring constant and drag coefficient of a flow-coupled bead by restraining it in an optical trap until it became strongly coupled to the actin, as indicated by a significant displacement within the trap (Figure 4D). Then, we released the bead from the optical trap and recorded the relaxation with a high speed camera (Figure 4E). Analysis of the bead motion within the optical trap provides *k*_*eff*_ and the relaxation gives the drag coefficient, *γ*_*eff*_. The force is given by the distance between the trap and the bead. The displacement is given by the distance between the bead and the position of an unrestrained bead, which we extrapolated from the steady motion of the released bead (blue dashed-line). The resulting force-displacement curve is given in Figure 4F. In contrast to a simple spring, the curve is non-linear with a slope varying from 25 pN/µm at small displacements to 165 pN/µm at larger displacements. The nonlinearity of this curve has many potential sources, but we expect that it is primarily dominated by bead rotation as discussed in the next section and the Supporting Material. Similarly, the slope of the curve relating applied force to bead velocity gives the drag coefficient. We analyzed the high-speed video of the released bead to obtain bead velocity and displacement, and use the force-displacement curve in Figure 4F to obtain the force. The resulting force-velocity curve is shown in Figure 4G. Over the accessible range, the effective drag coefficient is *γ*_*eff*_=0.8 pN∙s/μm. This drag coefficient is about 16 times larger than what a 2 µm bead would experience 100 nm away from a wall in water [30]. Thus, the drag experienced by the bead was dominated by the cell, not the surrounding fluid. Using this drag coefficient and the velocity of actin retrograde flow, we can estimate the magnitude of the viscous forces on restrained beads to be *γ*_*eff*_ *v*_*R*_≈ 0.1 pN. The displacements of a trapped bead under this force are indistinguishable from the fluctuating background. Much larger forces are readily generated by the deformation of the elastic element connecting the bead to the flowing actin.

Interestingly, the elastic element appears to undergo micron-scale deformations (Figure 4C and E). This suggests that the connection between the bead and the flowing actin is not a single-molecule, or even many molecules connected in parallel to the bead and actin. There are several candidates for elastic elements that could produce such large deformations under picoNewton forces, including the cell membrane and/or cross-linked polymer networks –each of which are considered below.

**Figure 5:**
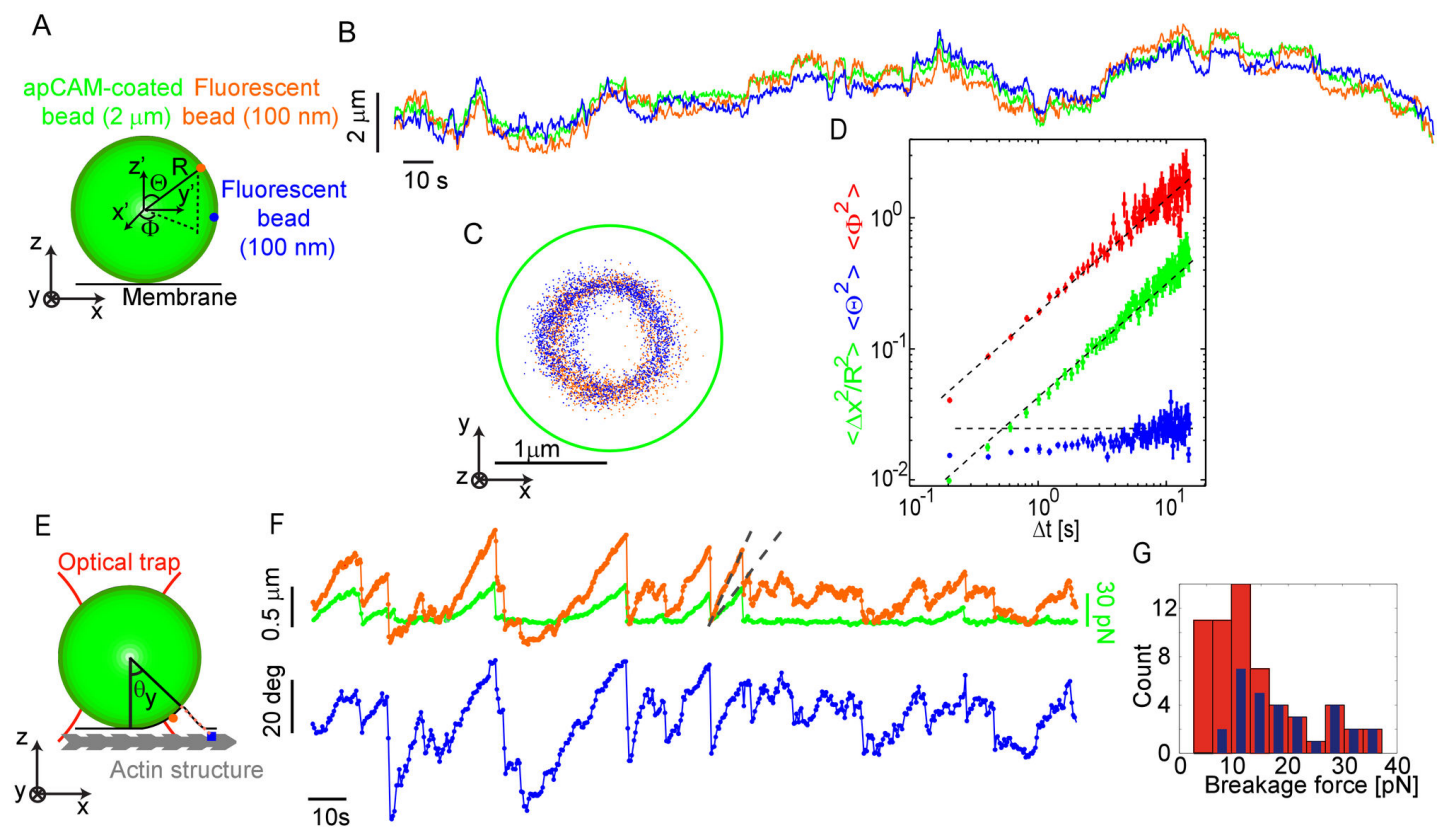
Membrane deforms but is too weak to explain observed traction forces. (A) Schematic representation of the setup: a 2 µm diameter apCAM-coated bead with two 100 nm fluorescent beads irreversibly attached on its surface is placed on the membrane of growth cone treated with 5 µM latrunculin B. (B) Displacement over time of the apCAM-coated bead (green) and the two fluorescent beads (red and blue) along the x-axis. (C) Positions in the x-y plane of the two fluorescent beads in the frame of reference of the center of mass of the apCAM-coated bead (green circle indicates the bead’s circumference). (D) Translational mean squared displacement of the apCAM-coated bead (green) and rotational mean squared displacements of the twisting angle (red) and rolling angle (blue). (E) Schematic representation of the setup: a 2 µm diameter apCAM-coated bead with a 100 nm diameter fluorescent bead irreversibly attached on its surface is placed on the membrane of a control growth cone. (F) Displacement over time of the apCAM-coated bead (green) and the small fluorescent bead (orange) along the actin flow. Green force scale bar applies only to the green trace. Rolling angle (blue) calculated from the small bead motion respect to the apCAM-coated bead motion. (G) Breakage force histograms calculated from centroids of the apCAM-coated bead (blue) and the fluorescent beads on its surface (red).

To characterize the relevant mechanical properties of the cell membrane, we measured the translational and rotational Brownian motion of apCAM-coated beads attached to growth cones treated with latrunculin (Figure 5A-D) [31]. Latrunculin binds to G-actin monomers and prevents them from polymerizing into the F-actin networks responsible for retrograde flow. With this treatment, we expect that the motion of a bead attached to the membrane is passive and determined by thermal fluctuations. Therefore, the kinematic parameters describing the observed Brownian motion can be related to dynamic properties of the bead/membrane complex, including the drag and spring coefficients, using the fluctuation-dissipation theorem. The translational motion of the large 2µm beads in the plane of the membrane was quantified using centroid tracking algorithms while their rotational Brownian motion was detected by imaging multiple smaller fluorescent beads (100 nm) attached to their surface, as outlined in the schematic Figure 5A.

A typical translational trajectory for a 2µm bead is shown as a green trace in Figure 5B. As expected in the absence of flowing actin the trajectory shows no hint of any steady translation. We quantified the trajectory by calculating the mean-squared displacement, ⟨Δ*x*(*t*)^2^⟩, as a function of lag time (green data points, Figure 5D). For simple Brownian motion, we expect ⟨Δ*x*(*t*)^2^⟩=2*Dt*. This has a slope of one on a log-log plot. The vertical offset gives the diffusion coefficient, *D*=0.018±0.001 μm^2^/s for the data in Figure 5D. The standard deviation across growth cones was 0.023 µm^2^/s. The mean-squared displacements for each growth cone are shown in Figure S4. Assuming that the bead/membrane complex was fluctuating near equilibrium in the absence of actin, we used the fluctuation-dissipation theorem to calculate the mean drag coefficient, *γ*_*eff*_=*k*_*B*_ *T*/*D* = 0.24 pN∙s/μm. This is about three times smaller than our measurement of *γ*_*eff*_ in Figure 4G, which was performed in the presence of unperturbed flowing actin.

These results are consistent with the well-established dynamics of a fluid membrane at these time and length scales [32]. Therefore, in-plane deformation of the membrane itself cannot explain the elastic element suggested by Figures 2, 3, and 4. However, even fluid membranes exert an elastic restoring force when they are bent [33]. Here, rotational Brownian motion of an apCAM-coated bead is expected to bend a membrane when the turnover of connections between the membrane and bead is slow compared to the timescale of Brownian motion.

To quantify possible elastic resistance to the large bead bending the membrane, we measured its rotational Brownian motion by imaging two surface attached smaller fluorescent beads in latrunculin (Movie S4). An example of the resulting trajectories is found in Figure 5B, where the three traces show the centroid of the large bead (green) as well as the centroids of the two small beads on its surface (red and blue). We re-plotted these trajectories in the frame of reference of the large bead in Figure 5C. The two small beads essentially diffused in a ring around the *z*-axis perpendicular to the cell membrane. This suggests that there is no elastic resistance to ‘twisting’ the bead around the *z*-axis in the absence of F-actin. However, the rotation along the *x*- and *y*-axes was greatly restricted, consistent with the membrane opposing ‘rolling’ of attached beads.

To quantify this rotational Brownian motion, we calculated the mean-squared displacements of the twist angle, *ϕ*, and the rolling angle, *θ* (the mathematical decomposition of the motion is discussed in the Supporting Material). We found that the twisting motion was nearly purely diffusive, with a slope of ⟨Δ*ϕ*(*t*)^2^⟩ near one on a log-log plot (Figure 5D, red trace). On the other hand, the rolling motion was strongly constrained (Figure 5D, blue trace). Another example showing the same trends is given in the Figure S5C. The rolling mean-squared displacement, ⟨Δ*θ*(*t*)^2^⟩, was nearly independent of time, as would be expected for Brownian motion on a spring. Specifically, ⟨Δ*θ*(*t*)^2^⟩ = 0.025 (horizontal dashed line, Figure 5D). Assuming again that these fluctuations were near equilibrium, we applied the equipartition theorem to extract a rotational spring constant, *η*=*k*_*B*_ *T*//⟨Δ*θ* ^2^⟩ = 0.16 pN·µm. Note that rolling and translation were independent of each other with an r^2^ value of 0.0452 for the correlation, as shown in Figure S5D.

To determine whether the bending of the membrane could generate the elastic restoring forces measured in Figure 4, we measured the rotational and translational motion of a trapped bead attached to a growth cone under control conditions with intact F-actin networks (Figure 5E schematic). The trajectory of the center of mass of the trapped bead and a fluorescent bead on its surface are shown in Figure 5F. Intriguingly, the small bead (orange trace) on the surface of the larger trapped bead (green trace) showed a qualitatively similar mix of steady translation and stationary fluctuations as seen for the center of mass in Figures 2-4. However, during periods of steady motion, the typical velocity at the surface was about 0.12 µm/s, significantly faster than the bead center, which translated at about 0.05 µm/s. This difference of speed between the surface and the center of the apCAM coated bead implies rotation, as can be clearly seen by a 3D rendering of this motion (Movie S5). Furthermore, when a connection between the bead and the cytoskeleton broke, the large bead not only translated back to the center of the optical trap, but also rotated to relieve the torque resulting from membrane bending.

To quantify this bead rotation, we calculated the rolling angle about the *y*-axis, *θ*_*y*_, which is parallel to the membrane and perpendicular to the flow direction (Figure 5E). We found that the angle of rotation increased as the flow coupled-bead moved away from the trap center and was as large as 60° (Figure 5F, blue trace). When the bead and membrane are well-adhered, the bead cannot roll without bending the membrane, creating a restoring torque. Using the rotational spring constant measured for latrunculin-treated growth cones, we find that the restoring torque’s contribution to the in-plane force cannot exceed *η*/*R* = 160 fN. This strongly suggests that membrane bending alone is insufficient to generate the picoNewton-scale elastic restoring forces observed in Figure 4.

While the displacements of the surface and center of the trapped bead were correlated, more coupling events could be resolved by observing the motion of the bead surface. Indeed, all measurable displacements of the bead center corresponded to displacements of its surface but not all of the resolvable displacements of the surface were visible in the trajectory of the bead center. Note examples in the traces (Figure 5F) where the bead either rotated prior to translation with retrograde flow or rotated without translocation. This suggests that previous studies characterizing the statistics of the breakage of bead-cytoskeleton linkages by tracking the translation of bead centers alone may have systematically undercounted breakage events. We demonstrate this in Figure 5G, where we compare breakage force histograms with events identified by bead translation (blue) or rotation (red). Evidently, the conventional approach missed about half of the events. The missed events are nearly all at low forces, where the mechanical response of the bead was dominated by rotation. This systematic bias would also cause the mean breakage force to be significantly over-estimated.

**Figure 6:**
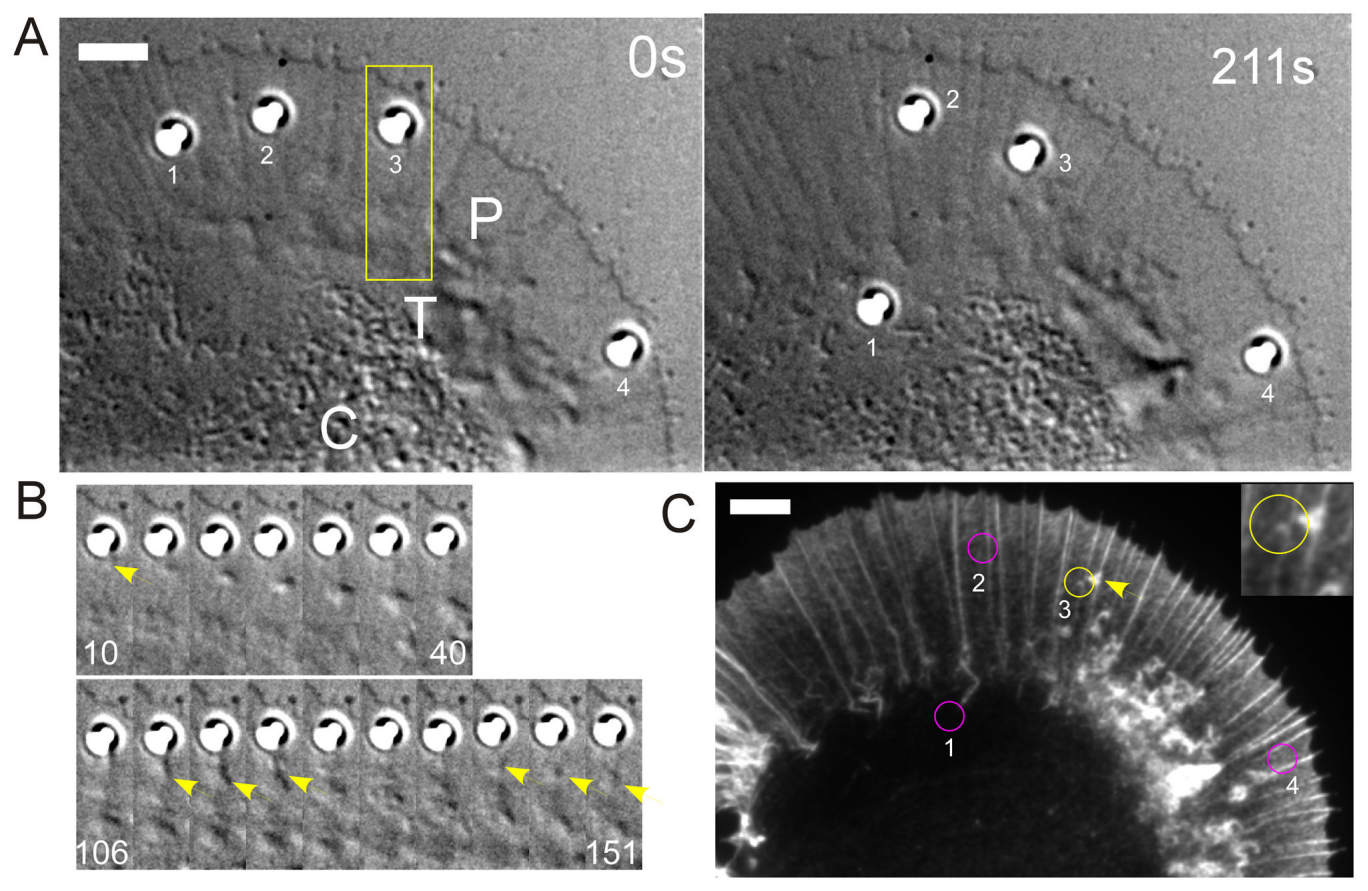
Actin filament assembly associated with optically restrained apCAM beads. (A) Four beads restrained with stationary optical traps. DIC images taken from the first (0s) and last (211s) frames of movie S6. Peripheral (P) domain, Transition (T) zone, and, central (C) domain. (B) Time-lapse montages depicting the region of interest (yellow box in (A)) at 5s intervals displayed at the same magnification. The beginning and end times (sec) are noted. Arrows indicate structures forming near the apCAM bead that travel with actin flow. The lower montage shows the bead immediately before fixation. (C) Corresponding phalloidin-stained actin filament structures. Final bead positions are indicated by circles. Yellow arrow indicates the actin structure associated with the bead shown in (B). Inset: 2x zoomed of bead 3. Scale bars, 5 µm.

Our results are consistent with a cross-linked network being responsible for the observed elasticity. An actin network with physical dimensions determined by the size of the bead could be reversibly stretched at the observed large deformations. Based upon the mechanical measurements in Figure S6; we estimate the elastic modulus of the nascent adhesion to be around 45-350 Pa, which is the right order of magnitude stiffness for a cross-linked actin network [34,35,36,37]. This actin could be part of the flowing actin network itself or new actin nucleated at the adhesion site [15,20,38,39]. We have previously reported that apCAM coated beads can trigger bouts of *de novo* actin assembly near bead binding sites [17]. Figure 6 illustrates examples of this type of behavior. Four beads were simultaneously restrained on the peripheral (P) domain using stationary (passive) optical traps (Figure 6A). During the 211 sec recording interval bead #1 escaped the optical trap and was transported by retrograde flow into the central (C) domain, beads 2-4 remained optically trapped in the P domain. Near bead #3 areas of increased refractive index typically associated with bursts of local actin assembly were observed (Figure 6B arrows, Movie S6). The growth cone was fixed immediately after the last time point shown in Figure 6B and labeled with fluorescent phalloidin. A cloudlike actin filament structure was observed near bead 3 (Figure 6C, inset).

## Conclusions

ApCAM is a homophilic Ig superfamily adhesion molecule related to vertebrate NCAM that can promote neurite outgrowth. Neurite outgrowth rates can be regulated by mechanical coupling of apCAM to underlying actin-network retrograde flow [15,27]. Here, we found that the coupling of apCAM to the actin flow can be dominated by either viscous drag or elastic energy storage, with roughly equal probability (Figures 3-4). Forces transmitted by viscous drag are in the order of 0.1 pN. Forces transmitted by the elastic connection can exceed 100 pN. The deformation of the elastic connection is reversible, and its failure under load is stochastic and catastrophic. Broadly interpreted, these results agree with the emerging three-stage picture of the development of adhesions [4,5], yet they provide new insight into the first two stages.

The first stage of adhesion maturation was previously described as an unengaged clutch, where the nascent adhesion site is not coupled to the flowing actin [5]. However, we find a persistent weak viscous drag ~0.1 pN is present (Figure 4). Larger forces are generated in the second stage. Here, there is an intermittent elastic coupling between the substrate and the underlying actin network. This coupling is typically modeled as a number of molecular springs in parallel, binding and unbinding to the substrate stochastically. Despite observation of elastic recoil events in a study of force-dependent integrin-cytoskeletal linkages [40], the molecular springs in general have been assumed to be rather brittle, breaking when stretched by no more than a few nanometers under a few picoNewtons of force [8,9,10,11,12,13]. This would cause the substrate to slip relative to the actin with a large apparent friction coefficient. In contrast to these models, we observe reversible elastic deformations approaching a micrometer. Such large deformations can only occur for a spatially extended compliant structure. There are several potential candidates, including the cell membrane, the adhesion complex and the cytoskeleton. Our measurements show that while the bending of the cell membrane can contribute to the elastic recovery, it does not appear to be strong enough to explain the magnitude of the observed forces. However, in the presence of actin, the membrane tension could be higher [41]. Recent measurements of the motion of the components of focal adhesions [42] observed differential velocities across the layers of focal adhesions. This relative motion was interpreted to be entirely due to slip, but it could also store some elastic energy. In principle, a preexisting and/or newly assembled actin network could store elastic energy consistent with our experimental findings. Note, however, that site-directed actin assembly could produce protrusive forces that are not accounted for in the simple mechanical model of Figure 3 [15]. Further studies addressing potential actin nucleation mechanisms involved will be reported elsewhere.

The seminal paper by Choquet et al. [40] focused on integrin-mediated stress dependent linkage reinforcement, but in fact their observations were also consistent with the fibronectin-cytoskeletal linkage possessing an elastic element. They observed cases were the bead "popped back" to its initial position after re-trapping and subsequent release (turning the trap off). This is very similar to our observations. Here, we directly tested the reversibility and mechanics of the elastic connection and did not directly test for reinforcement (Figures 2-4). In the cyclic trap-and-release protocol (Figure 2), the force used to pull the bead back was typically much greater than what the bead experienced in the stationary trap. We suspect that these forces broke any structure initially formed under the bead, masking potential weak reinforcement.

What are the benefits of a compliant connection between the substrate and the flowing actin? One possibility is that flexibility provides nascent adhesions with more time to reinforce before large forces develop. To appreciate this, consider a rigid substrate with a compliant connection to the flowing actin with a spring constant *k*_*eff*_. Under these conditions, the force on the adhesion increases at a rate equal to *k*_*eff*_ *v*_*R*_, where *v*_*R*_ is the retrograde flow speed. If the linker breaks at a characteristic force, *F*_*o*_, then the typical lifetime of the connection will be *τ*=*F*_*o*_/(*k*_*eff*_ *v*_*R*_). Thus, softer linkers can be stretched over a longer period of time without breaking. This would provide an opportunity for nascent adhesions to integrate mechanotransduction signals, thereby facilitating downstream signaling, recruitment and reinforcement [43,44,45,46].

## Supporting Information

Figure S1 **Effect of restraining time on transient size and frequency.** (*A*) Histogram of transient size for beads restrained in a fixed trap during 5 s (top histogram) and 20 s (bottom histogram). Inset: histograms of the steady state bead velocities after each transient. (*B*) Bar graph representing the percentage of flow coupled beads exhibiting no transient jump (blue) and exhibiting a jump (red) for 20 s, 10 s and 5 s restraining time in a fixed trap. (*C*) Mean transient (jump) size versus restraining time for events with no jump (blue) and events with jumps (red). Error bars represent the standard deviation. (*D*) Transient displacement versus applied force for 10 s restraining time with blebbistatin treatment. (*E*) Transient displacement versus applied force for 5 s (square) and 20 s (circle) restraining time in control conditions.
(TIF)

Figure S2 **Correlation of actin flow with bead dynamics.** (*A*) Example of a bead trajectory (green) superimposed on a kymograph of the underlying actin. Magenta lines indicate the retrograde flow rate. (*B*) Tracked-feature (in blue) and bead positions (in green) over time. In panels (*A*) and (*B*), the asterisks indicate the start of the bead translation away from the optical trap center and the arrow indicates the point of maximum force.
(TIF)

Figure S3 **Velocity of coupled beads does not depend on the clamping force.** Mean bead velocities are plotted against clamping force; each color series corresponds to one bead subjected to multiple trials in which the constant force level was varied in each trial. This raw data is depicted as a velocity-normalized plot in Figure 4C.
(TIF)

Figure S4 **Mean square displacement of apCAM-coated beads on the membrane of latrunculin-treated growth cones.** Black lines are fits to extract the diffusion coefficients.
(TIF)

Figure S5 **Measuring bead rotation.** (*A*) Histogram of the measured distance between two fluorescent 100 nm beads attached to the surface of a freely diffusing 2 µm bead. (*B*) Coordinate system for bead rotation experiments. (*C*) A second example of the mean-squared displacements of the translation (green), twisting (red) and rolling (blue) of a bead attached to a latrunculin treated growth cone. The inset shows the small bead trajectories in the frame of reference of the centroid of the large bead. (*D*) Scatter plot of rotational and translational displacements.
Using image analysis of our NIR brightfield images, we determine the centroid of the large bead in the x-y plane. We also find the centroids of small 100nm beads on its surface using fluorescent images. From this, we can calculate the positions of the small beads on the surface relative to the center of the big bead,${\vec{x}}_{i}$. While the x and y components are determined directly from the images, the z component can be determined using the fact that the beads of radius r are attached to a sphere of known radius, R. In that case, Pythagoras’ theorem tells us that${\left(R+r\right)}^{2}=x_{i}^{2}+y_{i}^{2}+z_{i}^{2}$. To quantify the tracking error, we generate a histogram of the separation of two small beads on the surface over many images, as shown in Figure S5. This gives us a tracking error of 12 nm.
With the coordinates of the large bead’s center and the small beads on its surface, we can measure the change in orientation of the large bead. Specifically, we calculate the rotation matrix, **R**(Δ*t*), describing the change in the large bead’s orientation over successive time points,${\vec{x}}_{i}(t+\Delta t)=R(\Delta t){\vec{x}}_{i}(t)$.
The rotation matrix can be expressed in terms of *θ*_*x*_, *θ*_*y*_ and *θ*_*z*_, the rotations about the x-axis, y-axis and z-axis, respectively.
$R=R_{x}R_{y}R_{z}$ (1),.
where$R_{x}$, $R_{y}$, $R_{z}$ are the rotation matrices about the x, y and z axis respectively.
To determine the values of the rotation angles, we use least squares minimization. Specifically, we vary *θ*_*x*_, *θ*_*y*_ and *θ*_*z*_ to minimize the sum.
$\sum_{i}{\left(R(\Delta t){\vec{x}}_{i}(t)-{\vec{x}}_{i}(t+\Delta t)\right)}^{2}$(2).
Rotation about the z-axis describes the ‘twisting’ of a bead attached to the cell membrane. Rotation about x y or any combination of the two corresponds to ‘rolling’ on the surface of the cell. To quantify the magnitude of rolling *θ*, irrespective of the orientation of the axis, we simply apply the rotation matrix to$\hat{z}$, which is unaffected by twist, $\cos\theta =\hat{z}\cdot R\hat{z}$.
(TIF)

Figure S6 **Effect of bead rotation on the force-extension curve of nascent adhesion.** (See supporting materials for discussion.) (*A*) Displacement over time of a flow-coupled apCAM-coated bead (green), a small 100nm fluorescent bead on its surface (orange) and the actin on which it attaches (blue). The trap was turned off when the bead-trap displacement reached 0.7 µm and re-trapped. (*B*) Rolling angle of the apCAM-coated bead over time. The black points correspond to the prediction of our simple torque balance model. (*C*) Calculation of force-extension curve of the nascent adhesion. Top, schematic of rotating bead. R, bead radius, s, extension of connection, xB, bead center, xA, position of nascent adhesion to actin. Lower panel, uncorrected force extension curve based on bead centroid position (blue) and rotation-corrected force-extension curve (red).
To demonstrate the impact of bead rotation on the force-extension curves, we reproduce the protocol shown in Figure 3 D while measuring the bead rotation (Figure S6 A). As the large apCAM-coated bead couples to the actin flow, the small bead initially moves at the retrograde flow rate without any resolvable translation of the large bead (Figure S6 A). The resulting angular displacements are shown in Figure S6 B. When the bead initially couples to the flowing actin, it rotates without translation. Once larger forces are attained, rotation stops and translation dominates.
We compare the measured rotation to that expected by a simple torque balance on the large bead. If the force transmitted from the flowing actin to the bead is dominated by a connection at a single point, the angle of rotation, *θ*_*y*_ is given by *sin*(*θ_y_*)=(*x*_*A*_-*x*_*B*_)/(*R*+*s*). Here *x*_*B*_ is the center of the large bead and *x*_*A*_ is the position where the nascent adhesion is attached to the flowing actin. The extension, s, of the connection between the bead and actin is giving by$s=\sqrt{{\left(x_{A}-x_{B}\right)}^{2}+R^{2}}-R$. We find a good agreement between the measured and predicted angles, which are shown as a black points in Figure S6 B. We do not always see good agreement between the measured rotation and this simple torque balance, suggesting that the bead can couple to the actin through multiple points.
In the event that the rotation is well described by a connection at a single point, the extension is well defined and we can construct a force-extension curve. Assuming that the in-plane force is the projection of a force,*F*, along the connection, we have *F*=*F*_*trap*_/*sin*(*θ_y_*). The resulting force curve is shown in red alongside the apparent force-extension curve based solely on bead translation in blue, Figure S6 C. The rotation corrected force-extension curve is much more linear. The estimated stiffness of the nascent adhesion is the slope of the corrected force-extension curve, 45 pN/µm. This suggests an underlying material with an elastic modulus from 45 to 300 Pa, using characteristic length scales from 150 nm, the characteristic thickness of the lamellipodium [47], to 1000 nm, the radius of the bead.
(TIF)

Movie S1 **Multi-mode imaging of optically restrained apCAM-bead.** Growth cone structure shown in DIC; actin filament structure shown by fluorescent phalloidin labeling. Field of view: 80 µm x 50 µm. 268 frames (elapsed time 670 s).
(MP4)

Movie S2 **DIC time-lapse showing the trap and release assay.** Successive holding time of 10, 5 and 20 s shown. Optical trap is indicated by red cross. Field of view: 20 µm x 20 µm. 1684 frames (elapsed time 1370 s).
(AVI)

Movie S3 **DIC time-lapse showing the force feedback assay.** Bead contour is highlighted in green, optical trap is indicated by the red cross. Field of view: 42 µm x 42 µm. 153 frames (elapsed time 153 s).
(MP4)

Movie S4 **Animation of recorded bead motion for a latrunculin-treated growth cone.** Rendering of 2µm apCAM-coated bead (green) and 100 nm fluorescent bead (red and blue) attached to its surface. Left panel: side view, right panel: bottom view. 798 frames (elapsed time 164 s).
(AVI)

Movie S5 **Animation of recorded bead motion for a control growth cone.** Rendering of 2µm apCAM-coated bead motion (green) and 100 nm fluorescent bead motion (red) attached on its surface on the membrane of a control growth cone. Left panel: side view, right panel: bottom view. 491 frames (elapsed time 210 s).
(AVI)

Movie S6 **DIC time-lapse showing four apCAM beads restrained in stationary optical traps on the P domain of an *Aplysia* growth cone.** Yellow arrows in movie indicate refractive structures forming near one of the beads that travel with retrograde flow. Field of view: 40 µm x 33 µm (elapsed time 237 sec).
(WMV)
