# Supplementary figures and images for: Elastic Coupling of Nascent apCAM Adhesions to Flowing Actin Networks
Source: PLoS One. 2013 Sep 6;8(9):e73389. doi: 10.1371/journal.pone.0073389 (PMC3765355; doi:10.1371/journal.pone.0073389)

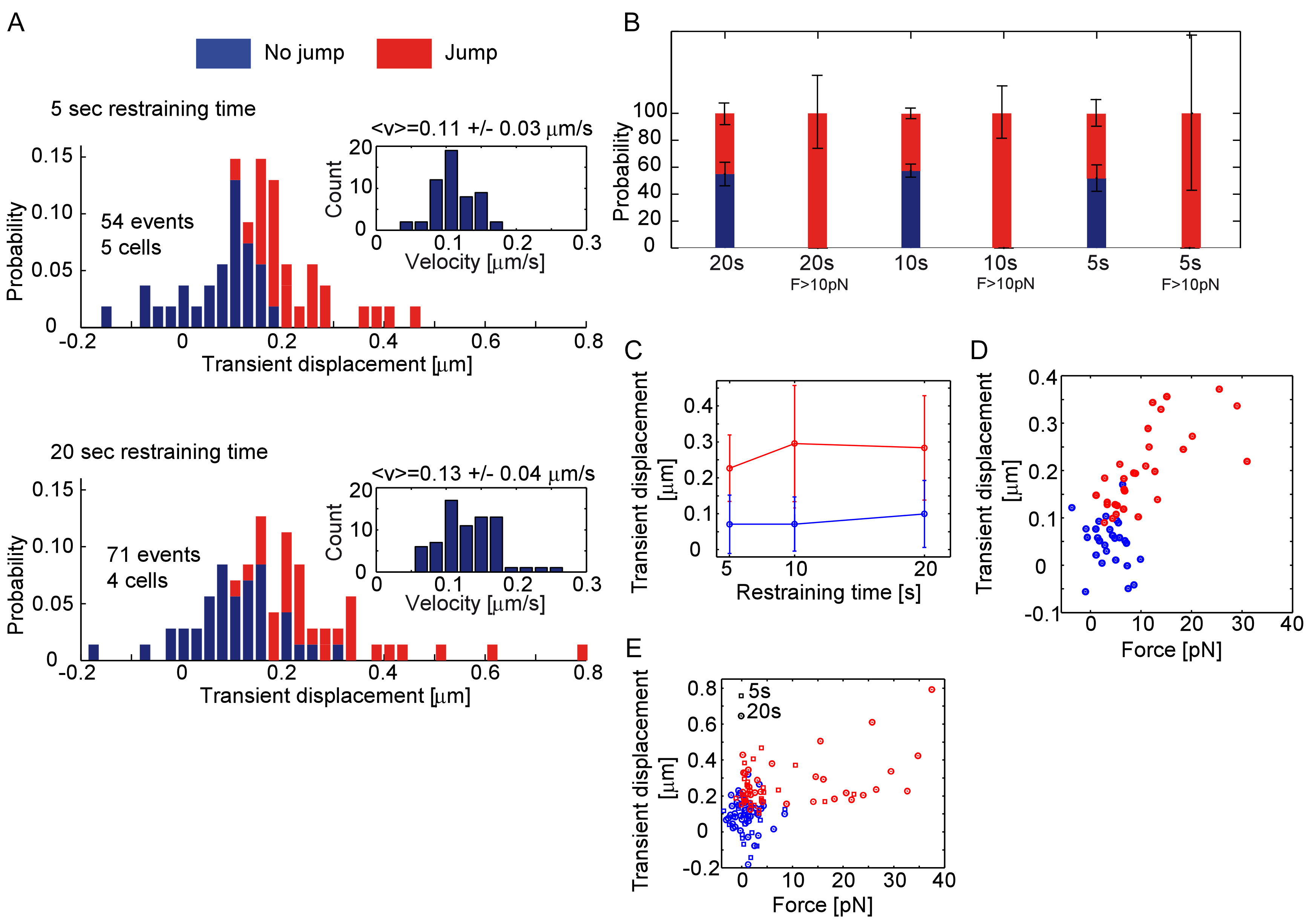

Supplement: Figure S1 — Effect of restraining time on transient size and frequency. (A) Histogram of transient size for beads restrained in a fixed trap during 5 s (top histogram) and 20 s (bottom histogram). Inset: histograms of the steady state bead velocities after each transient. (B) Bar graph representing the percentage of flow coupled beads exhibiting no transient jump (blue) and exhibiting a jump (red) for 20 s, 10 s and 5 s restraining time in a fixed trap. (C) Mean transient (jump) size versus restraining time for events with no jump (blue) and events with jumps (red). Error bars represent the standard deviation. (D) Transient displacement versus applied force for 10 s restraining time with blebbistatin treatment. (E) Transient displacement versus applied force for 5 s (square) and 20 s (circle) restraining time in control conditions. (TIF) [file pone.0073389.s001.tif]

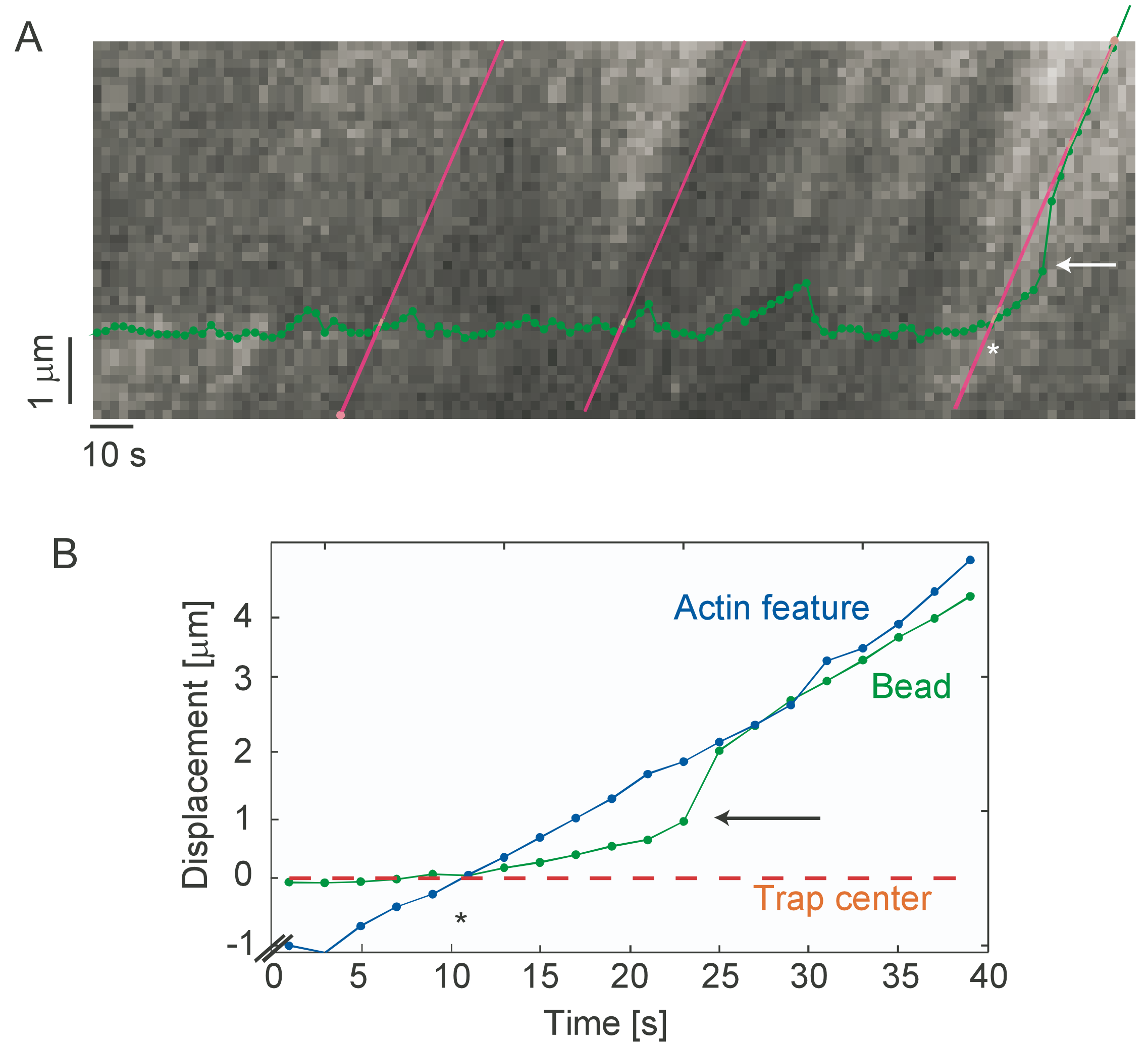

Supplement: Figure S2 — Correlation of actin flow with bead dynamics. (A) Example of a bead trajectory (green) superimposed on a kymograph of the underlying actin. Magenta lines indicate the retrograde flow rate. (B) Tracked-feature (in blue) and bead positions (in green) over time. In panels (A) and (B), the asterisks indicate the start of the bead translation away from the optical trap center and the arrow indicates the point of maximum force. (TIF) [file pone.0073389.s002.tif]

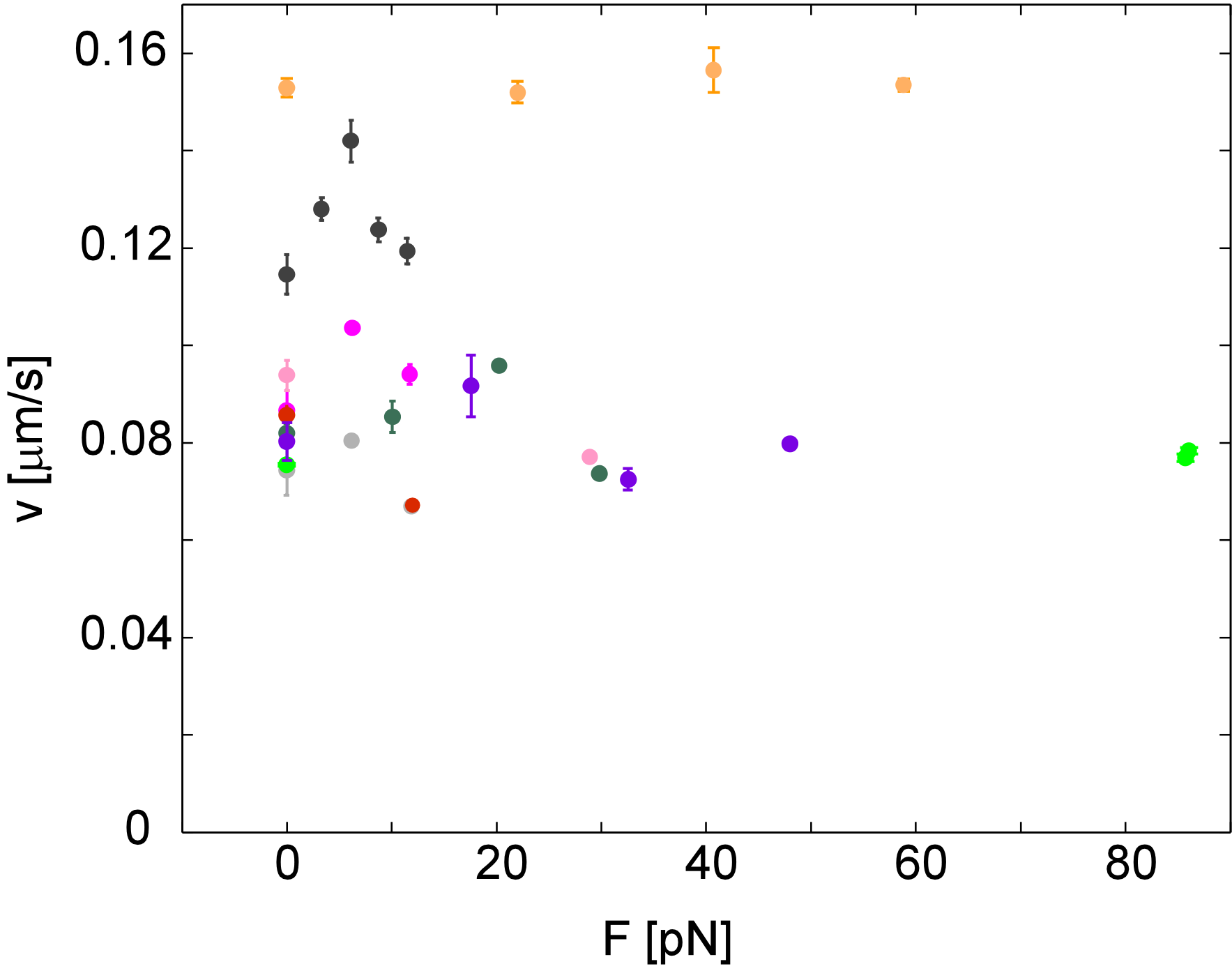

Supplement: Figure S3 — Velocity of coupled beads does not depend on the clamping force. Mean bead velocities are plotted against clamping force; each color series corresponds to one bead subjected to multiple trials in which the constant force level was varied in each trial. This raw data is depicted as a velocity-normalized plot in Figure 4C. (TIF) [file pone.0073389.s003.tif]

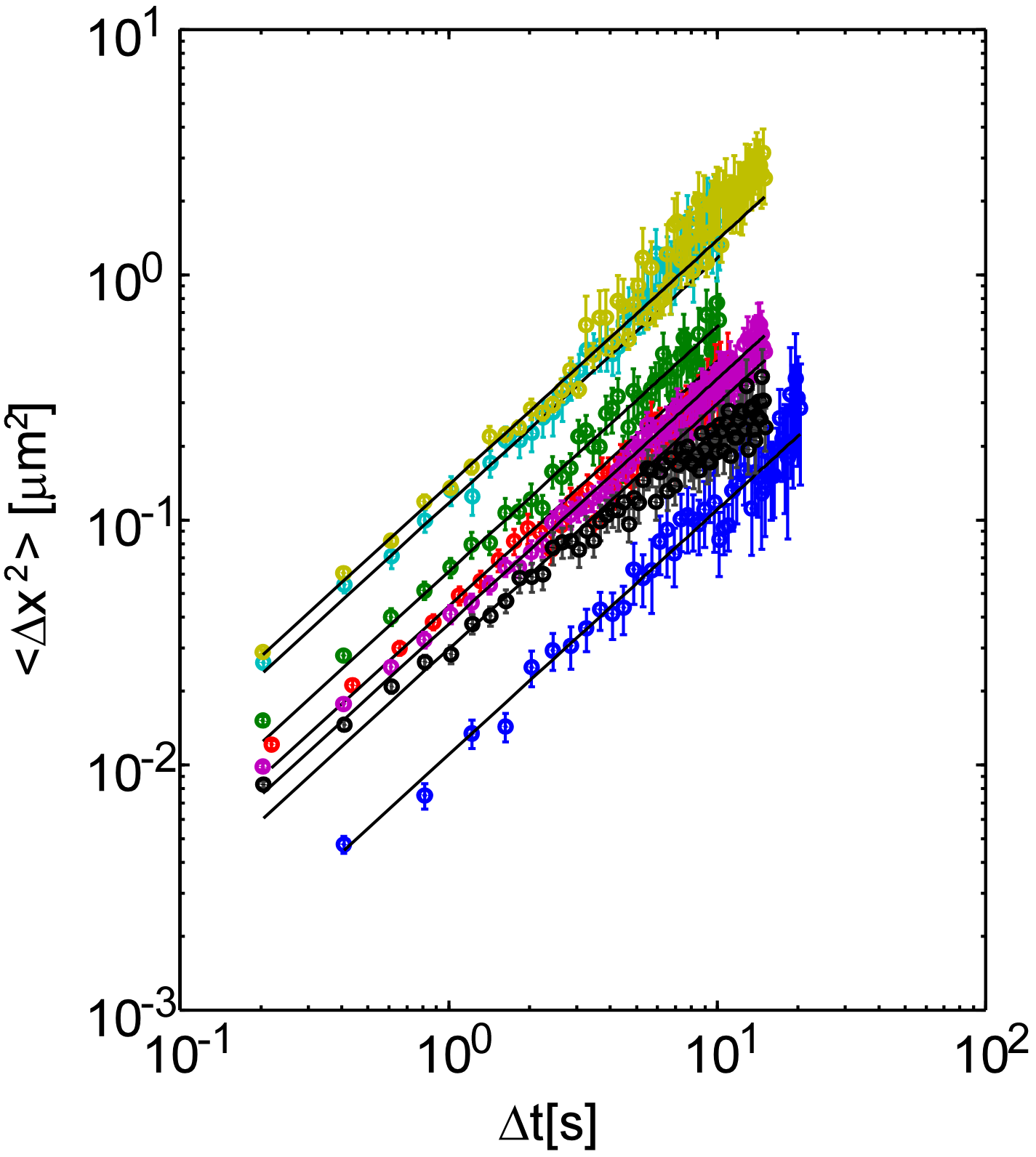

Supplement: Figure S4 — Mean square displacement of apCAM-coated beads on the membrane of latrunculin-treated growth cones. Black lines are fits to extract the diffusion coefficients. (TIF) [file pone.0073389.s004.tif]

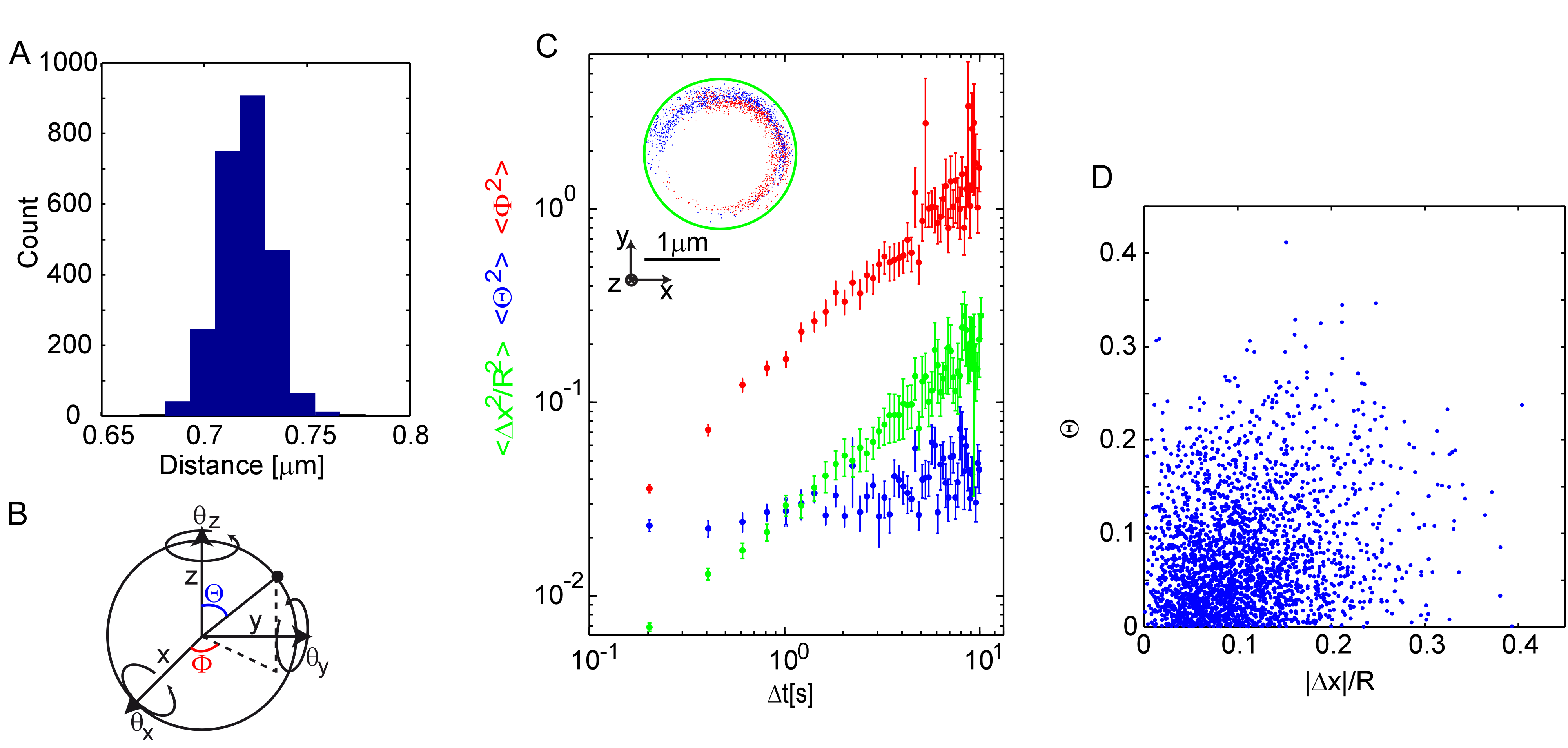

Supplement: Figure S5 — Measuring bead rotation. (A) Histogram of the measured distance between two fluorescent 100 nm beads attached to the surface of a freely diffusing 2 µm bead. (B) Coordinate system for bead rotation experiments. (C) A second example of the mean-squared displacements of the translation (green), twisting (red) and rolling (blue) of a bead attached to a latrunculin treated growth cone. The inset shows the small bead trajectories in the frame of reference of the centroid of the large bead. (D) Scatter plot of rotational and translational displacements. Using image analysis of our NIR brightfield images, we determine the centroid of the large bead in the x-y plane. We also find the centroids of small 100nm beads on its surface using fluorescent images. From this, we can calculate the positions of the small beads on the surface relative to the center of the big bead,x→i. While the x and y components are determined directly from the images, the z component can be determined using the fact that the beads of radius r are attached to a sphere of known radius, R. In that case, Pythagoras’ theorem tells us that(R+r)2=xi2+yi2+zi2. To quantify the tracking error, we generate a histogram of the separation of two small beads on the surface over many images, as shown in Figure S5. This gives us a tracking error of 12 nm. With the coordinates of the large bead’s center and the small beads on its surface, we can measure the change in orientation of the large bead. Specifically, we calculate the rotation matrix, R(Δt), describing the change in the large bead’s orientation over successive time points,x→i(t+Δt)=R(Δt)x→i(t). The rotation matrix can be expressed in terms of θ x, θ y and θ z, the rotations about the x-axis, y-axis and z-axis, respectively. R=RxRyRz (1),. whereRx, Ry, Rz are the rotation matrices about the x, y and z axis respectively. To determine the values of the rotation angles, we use least squares minimization. Specifically, we vary θ x, θ y and θ z to minimize the sum. [file pone.0073389.s005.tif]

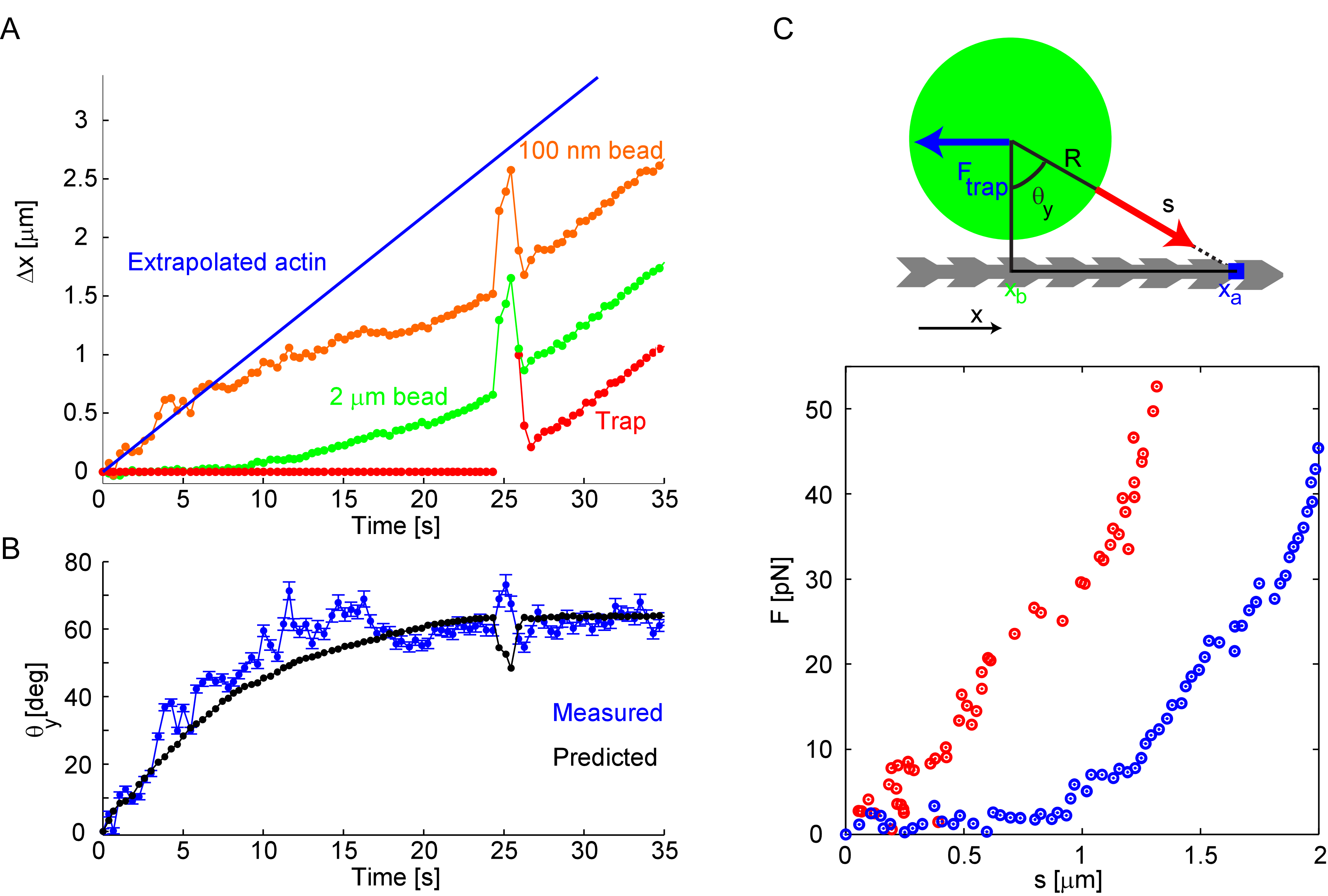

Supplement: Figure S6 — Effect of bead rotation on the force-extension curve of nascent adhesion. (See supporting materials for discussion.) (A) Displacement over time of a flow-coupled apCAM-coated bead (green), a small 100nm fluorescent bead on its surface (orange) and the actin on which it attaches (blue). The trap was turned off when the bead-trap displacement reached 0.7 µm and re-trapped. (B) Rolling angle of the apCAM-coated bead over time. The black points correspond to the prediction of our simple torque balance model. (C) Calculation of force-extension curve of the nascent adhesion. Top, schematic of rotating bead. R, bead radius, s, extension of connection, xB, bead center, xA, position of nascent adhesion to actin. Lower panel, uncorrected force extension curve based on bead centroid position (blue) and rotation-corrected force-extension curve (red). To demonstrate the impact of bead rotation on the force-extension curves, we reproduce the protocol shown in Figure 3 D while measuring the bead rotation (Figure S6 A). As the large apCAM-coated bead couples to the actin flow, the small bead initially moves at the retrograde flow rate without any resolvable translation of the large bead (Figure S6 A). The resulting angular displacements are shown in Figure S6 B. When the bead initially couples to the flowing actin, it rotates without translation. Once larger forces are attained, rotation stops and translation dominates. We compare the measured rotation to that expected by a simple torque balance on the large bead. If the force transmitted from the flowing actin to the bead is dominated by a connection at a single point, the angle of rotation, θ y is given by sin(θy)=(x A-x B)/(R+s). Here x B is the center of the large bead and x A is the position where the nascent adhesion is attached to the flowing actin. The extension, s, of the connection between the bead and actin is giving bys=(xA−xB)2+R2−R. We find a good agreement between the measured and predicted angles, which are shown as [file pone.0073389.s006.tif]
